# Splice Variants of Activation Induced Deaminase (AID) Do Not Affect the Efficiency of Class Switch Recombination in Murine CH12F3 Cells

**DOI:** 10.1371/journal.pone.0121719

**Published:** 2015-03-24

**Authors:** Cesare Sala, Giorgio Mattiuz, Silvia Pietrobono, Andrea Chicca, Silvestro G. Conticello

**Affiliations:** 1 Core Research Laboratory—Istituto Toscano Tumori, Firenze, Italy; 2 Department of Medical Biotechnologies, University of Siena, Siena, Italy; 3 Department of Oncology—Azienda Ospedaliero-Universitaria Careggi, Firenze, Italy; Chang Gung University, TAIWAN

## Abstract

Activation Induced Deaminase (AID) triggers the antigen-driven antibody diversification processes through its ability to edit DNA. AID dependent DNA damage is also the cause of genetic alterations often found in mature B cell tumors. A number of splice variants of AID have been identified, for which a role in the modulation of its activity has been hypothesized. We have thus tested two of these splice variants, which we find catalytically inactive, for their ability to modulate the activity of endogenous AID in CH12F3 cells, a murine lymphoma cell line in which Class Switch Recombination (CSR) can be induced. In contrast to full-length AID, neither these splice variants or a catalytically impaired AID mutant affect the efficiency of Class Switch Recombination. Thus, while a role for these splice variants at the RNA level remains possible, it is unlikely that they exert any regulatory effect on the function of AID.

## Introduction

Activation Induced Deaminase (AID) is the DNA editing enzyme that begins the antigen driven diversification processes in activated B cells through its ability to deaminate DNA [1–3]. Whereas AID action is physiologically exerted on the immunoglobulin locus, AID dependent damage can induce mutations and chromosomal translocations in a cohort of other loci. Evidence for this comes from genetic analysis of tumors originating from mature B cells [4–6] as well as from experimental systems [7–13]. A number of regulatory safeguards limits the effects of AID in B cells in order to keep under control its potential damaging effects, from transcription [14,15], to cellular localization [16–23] and posttranslational modifications [24–26].

**Fig 1:**
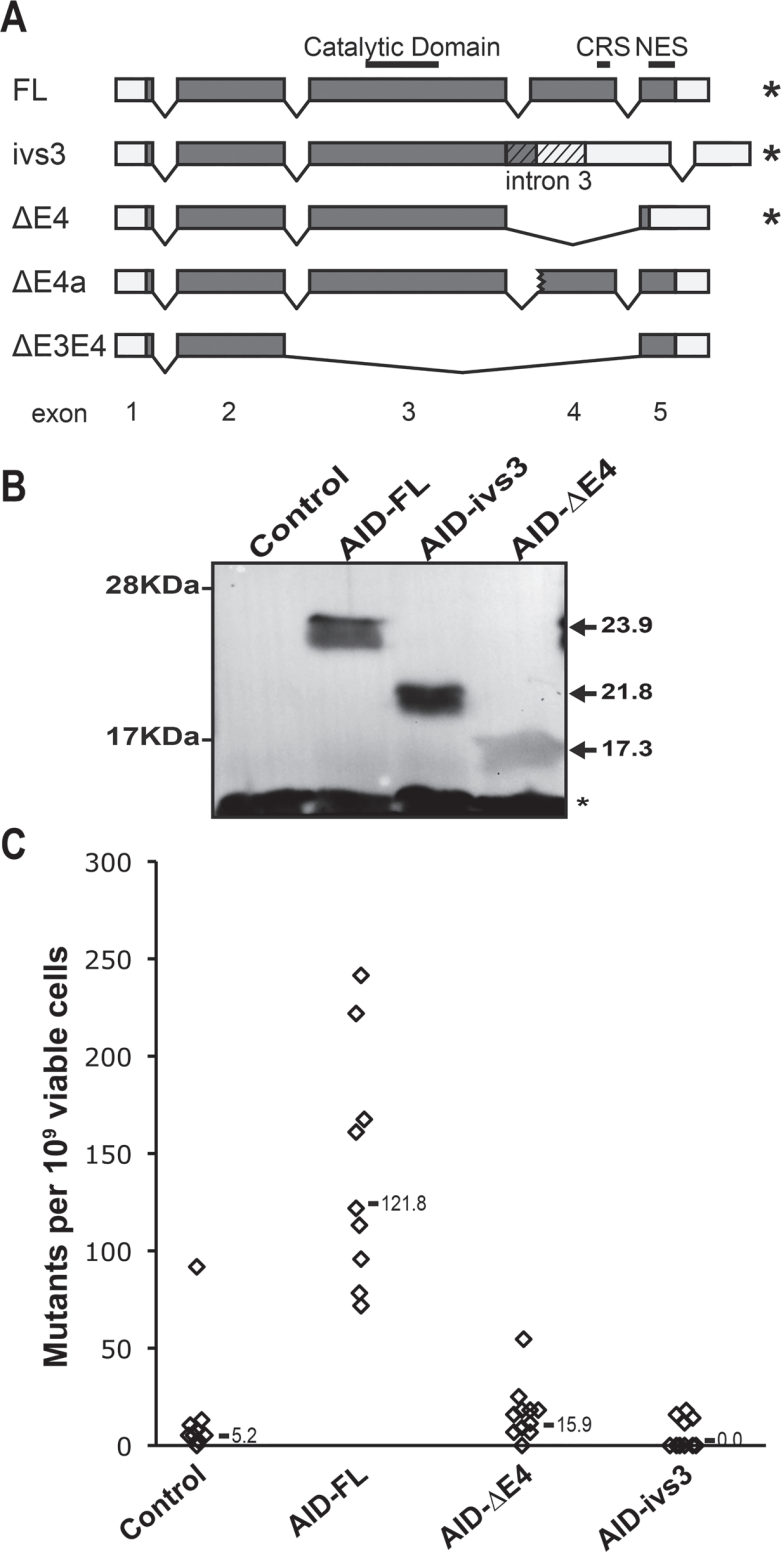
Schematic representation of the splice variants of AID and their activity in bacteria. (A) The exonic structure of the splice isoforms is shown. The position of functional features of the full-length AID (AID-FL) is indicated: the catalytic domain, the cytoplasmic retention signal (CRS; [20]) and the nuclear export signal (NES; [16–19]). The coding sequence appears in grey and the retained intron 3 is indicated (AID-ivs3) by the angle-striped pattern. The asterisks indicate the AID isoforms tested in the study. (B) Western blot analysis showing the expression levels of AID-FL, AID-ΔE4, AID-ivs3, or an empty plasmid in KL16 bacteria after induction with IPTG. Equal amounts of protein lysates (10 μg) were loaded on SDS-PAGE. The apparent molecular weights from prestained protein ladder are shown on left. The arrows indicate the expected molecular weight of the AID isoforms. The asterisk indicates an unspecific band. The asterisk indicates an unspecific band. (C) Rifampicin assay using the various AID isoforms. Only the revertants resistant to rifampicin can grow. While AID induces a mutator phenotype (*P*<10^−3^ by Dunn’s multiple comparison test), the tested splice variants display levels of revertants similar to the negative control (empty plasmid). The mutation rate is calculated after normalization with Ampicillin resistant viable colonies. The median value is indicated.

Expression of AID has been found in a number of B cell tumors [27–36]. Together with the full-length form of AID, other splice transcripts have been identified in B cell tumors, initially, and then in normal B cells [27–35,37,38] (Fig. 1A). Indeed, the presence of the various splice variants has been inversely correlated to the mutational status of the immunoglobulins in B-cell chronic lymphocytic leukemia [27,29–31,35]. This has opened the possibility that at least some of these splice variants might be part of the regulatory network of AID.

While initially deemed as catalytically active [34], subsequent reports clarified that AID splice variants are neither able to support deamination of DNA nor to trigger antibody diversification on their own [39,40]. Yet, even an inactive isoform could play a role in the regulation of AID: interaction with the catalytically active AID itself or with other molecules in its pathway could modify the physiologic activity of AID. In order to clarify this point we have thus tested the ability of some of these splice isoforms to affect the efficiency of Class Switch Recombination (CSR) in CH12F3 cells, a murine lymphoma cellular model in which the efficiency of CSR can be assessed [41].

## Materials and Methods

### Plasmids

The coding sequences of AID splice isoforms were obtained by RT-PCR (primer forward, AAAAAGCTTACCATGGACAGCCTCTTGATG; reverse, TTTCTCGAGTCAAAGTCCCAAAGTACGAAATG) from an RNA sample of B cell chronic lymphocytic leukemia (kindly provided by Dr. F. Forconi) and cloned either into the AID encoding pTrc99A plasmid for bacterial expression ([2]; SnaBI and XhoI sites) or in the pAID-express-puro2 plasmid ([42]; primer forward, AAAGCTAGCACCATGGACAGCCTCTTGATG and reverse, AAAGCTAGCACCATGGACAGCCTCTTGATG; BglII and NheI sites), in which the ß-actin promoter drives the expression of AID in mammalian cells alongside an EGFP reporter gene, linked to the AID transcript through an internal ribosome entry site (IRES). The catalytically inactive AID E58A mutant was prepared by site directed mutagenesis on the AID encoding construct using the primers forward, CTGCCACGTGGCATTGCTCTTCCTCCGC and reverse, GGAAGAGCAATGCCACGTGGCAGCCGTT. The mutator activity of the splice variants was assayed in a bacterial assay in which the frequency of revertant colonies is measured after rifampicin treatment [2].

### Cells

HEK293T cells [43] were maintained in DMEM supplemented with 10% FBS, 2mM L-Glutamine, and penicillin/streptomycin at 37°C in 5% CO_2_. Transient transfection was performed using X-tremeGene HP DNA transfection reagent (Roche Diagnostics, Basel, Switzerland) according to manufacturer’s instructions.

CH12F3 cells (kindly provided by Eva Severinson and Tasuku Honjo) [41] were maintained in RPMI1640 supplemented with 10% FBS, 50 μM ß-mercaptoethanol, 2mM L-Glutamine, 1mM Sodium pyruvate and penicillin/streptomycin at 37°C in 5% CO_2_. The plasmids have been transfected in CH12F3 cells by electroporation using a Gene Pulser II electroporator (Biorad, Hercules, CA) (Voltage = 250V; Capacity = 500 μF; Resistance = ∞.) and independent stable clones were selected through puromycin selection (0.6 μg/ml). Selected clones were then screened for EGFP expression (coexpressed with AID via an IRES) by flow cytometry.

### Analysis of Class Switch Recombination

Class Switch Recombination in CH12F3 cells was induced with TGF-ß (2 ng/ml), IL4 (2 μg/ml) and anti-CD40 antibody (0.5 mg/ml) as described in Nakamura et al. [41], in medium containing 30% FBS. After 72 hours in culture CSR was assayed in stimulated cells by FACS using an anti-IgA antibody conjugated with RPE (Southern Biotech, Birmingham, AL; 1:100). Flow cytometry analysis was performed on a Accuri C6 flow cytometer with a standard configuration (BD Biosciences, San Jose, CA).

### Analysis of the expression levels

The protein levels of AID variants from induced bacteria or transiently transfected HEK293T cells were assayed by western blot analysis after lysis with RIPA buffer and SDS-PAGE. A monoclonal antibody raised against AID N-terminus was used (clone 52–1; 1:1000; [44]). ß-Actin was used as loading control (monoclonal Ab, clone AC-15, lot 117K4873, Sigma, St Louis, MI, USA; 1:10000).

Quantitative real-time PCR (qPCR) was carried out to analyze the transcription level of endogenous and exogenous AID in CH12F3 cells. Total RNA was isolated with TriPure Isolation Reagent (Roche Diagnostics) and subjected to DNase I treatment (Roche Diagnostics). Reverse transcription was performed with High Capacity cDNA Reverse Transcription Kit (Life Technologies, Paisley, UK). The qPCR reactions were performed at 60°C using SsoFastTM EvaGreen Supermix (Bio-Rad, Hercules, CA, USA) on a Rotorgene-Q (Qiagen) and analyzed using β-Actin as housekeeping gene. Primer sequences are: murine AID, CTCCTGCTCACTGGACTTCG (f), AGGCTGAGGTTAGGGTTCCA (r); human AID, CAGCCTCTTGATGAACCGGA (f), CGTGGCAGCCGTTCTTATTG (r); murine β-Actin, GGCTCCTAGCACCATGAAG (f), GAAAGGGTGTAAAACGCAGC (r).

### Statistical analysis

All statistical analyses were performed using Prism (Graphpad, La Jolla, CA USA). The Kruskal Wallis Test with *post hoc* Dunn's multiple comparison test was used for the rifampicin assay. One-way ANOVA with Tukey’s multiple comparison test was performed to analyze the Class Switch Recombination.

## Results and Discussion

We cloned two of the splice isoforms of AID (AID-ivs3 and AID-ΔE4) that encode for truncated forms of AID in which either the fourth exon is skipped or the fourth intron is retained (Fig. 1A). In either case such alternative splicing results in the lack of the C-terminal 57 amino acids of the catalytically active AID. These isoforms lack both the Nuclear Export Signal, necessary for an efficient CSR, and a cytoplasmic retention signal [16–20]. After cloning these splice variants in bacterial expression vectors (Fig. 1B), we tested their ability to induce a mutator phenotype in bacteria in the rifampicin-resistance reversion assay [2]. The inability of the splice variants of AID to induce an increased level of revertants suggests that they are catalytically inactive, as suggested by the previous *in vitro* analyses [39,40] (Fig. 1C).

**Fig 2:**
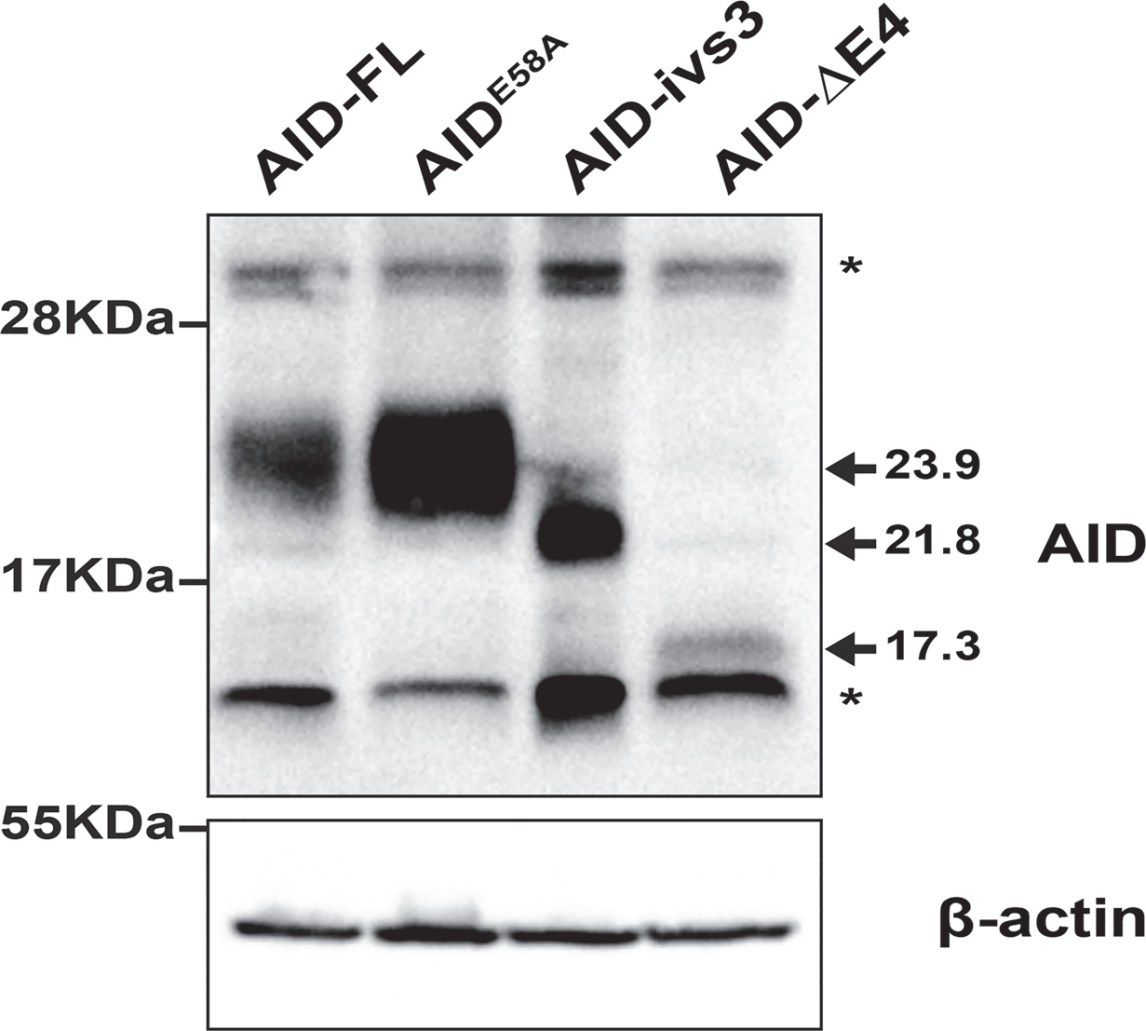
Expression of the AID variants in HEK293T cells. Western blot analysis of HEK293T cells transiently transfected with the various constructs (AID-FL, AID-ΔE4, AID-ivs3, AID^E58A^). Equal amounts of protein lysates (30 μg) were loaded on SDS-PAGE. ß-actin was used as a loading control. The apparent molecular weights from prestained protein ladder are shown on left. The arrows indicate the expected molecular weight of the AID isoforms. The asterisks indicate unspecific bands.

In order to assess the effects of these splice variants on Class Switch Recombination, we cloned them in a mammalian expression vector and we tested them in HEK293T cells. Compared to the full-length AID, the AID-ΔE4 variant consistently appeared expressed at lower levels, whereas the AID-ivs3 variant, which differs in its C-terminus from AID-ΔE4, was expressed at slightly higher levels (Fig. 2). This result is compatible with the increased instability of the splice variants observed by Rebhandl et al. [45].

**Fig 3:**
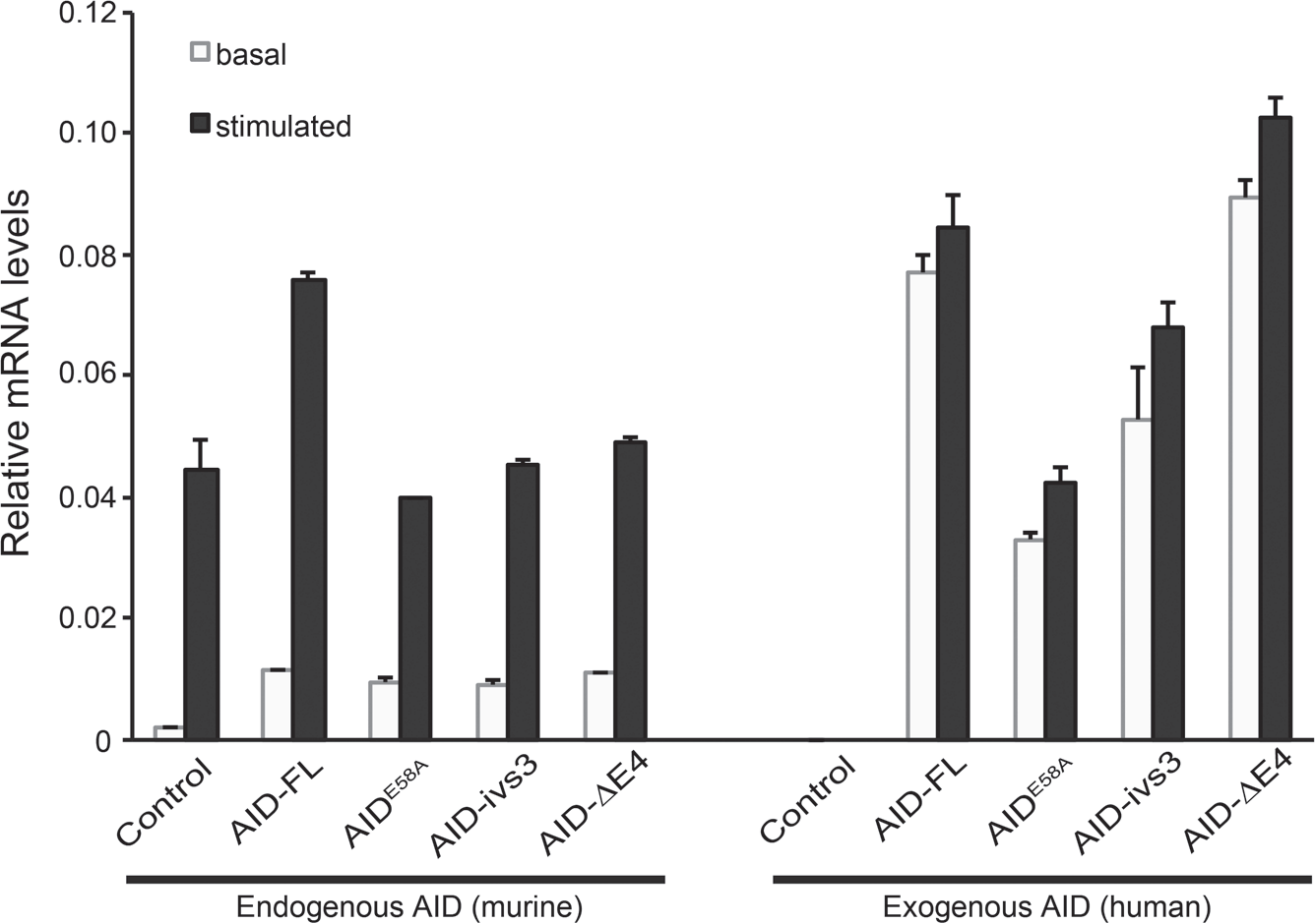
Expression of the AID variants in CH12F3 cells. Quantitative real-time PCR showing the expression levels of endogenous (murine) and exogenous (human) AID from representative clones of CH12F3 cells stably transfected with the constructs for AID-FL, AID-ΔE4, AID-ivs3, AID^E58A^, or from control cells. Basal expression levels (gray) are compared to those in stimulated cells (dark colums).

We next transfected all the mammalian expression constructs in CH12F3 cells, and we have selected clones expressing them by puromycin-selection and expression of the EGFP reporter. Quantitative real-time PCR of transfected CH12F3 cells before and after induction with TGF-ß, IL-4 and CD40 stimulation suggests that expression of the exogenous AID variants, at least at the transcriptional level, matches that of endogenous AID in stimulated cells (Fig. 3). Due to technical limitations, it is not possible to assess AID protein levels in CH12F3 cells, but it is reasonable to assume that protein levels of AID splice variants should be proportional to those observed in HEK293T cells and they might be preferentially degraded through the proteasome [45]. On the other hand, it must be taken into account that—contrarily to full-length AID—the splice variants are predominantly nuclear [39]. The probable stoichiometric ratio between wild-type and splice variant would not support a direct interaction between the isoforms. Nonetheless, even though steady-state levels of the splice variants could be lower than those of endogenous AID in stimulated CH12F3 cells, it could still be possible that the splice variants could interfere with the physiological activity of AID through the catabolic pathway.

**Fig 4:**
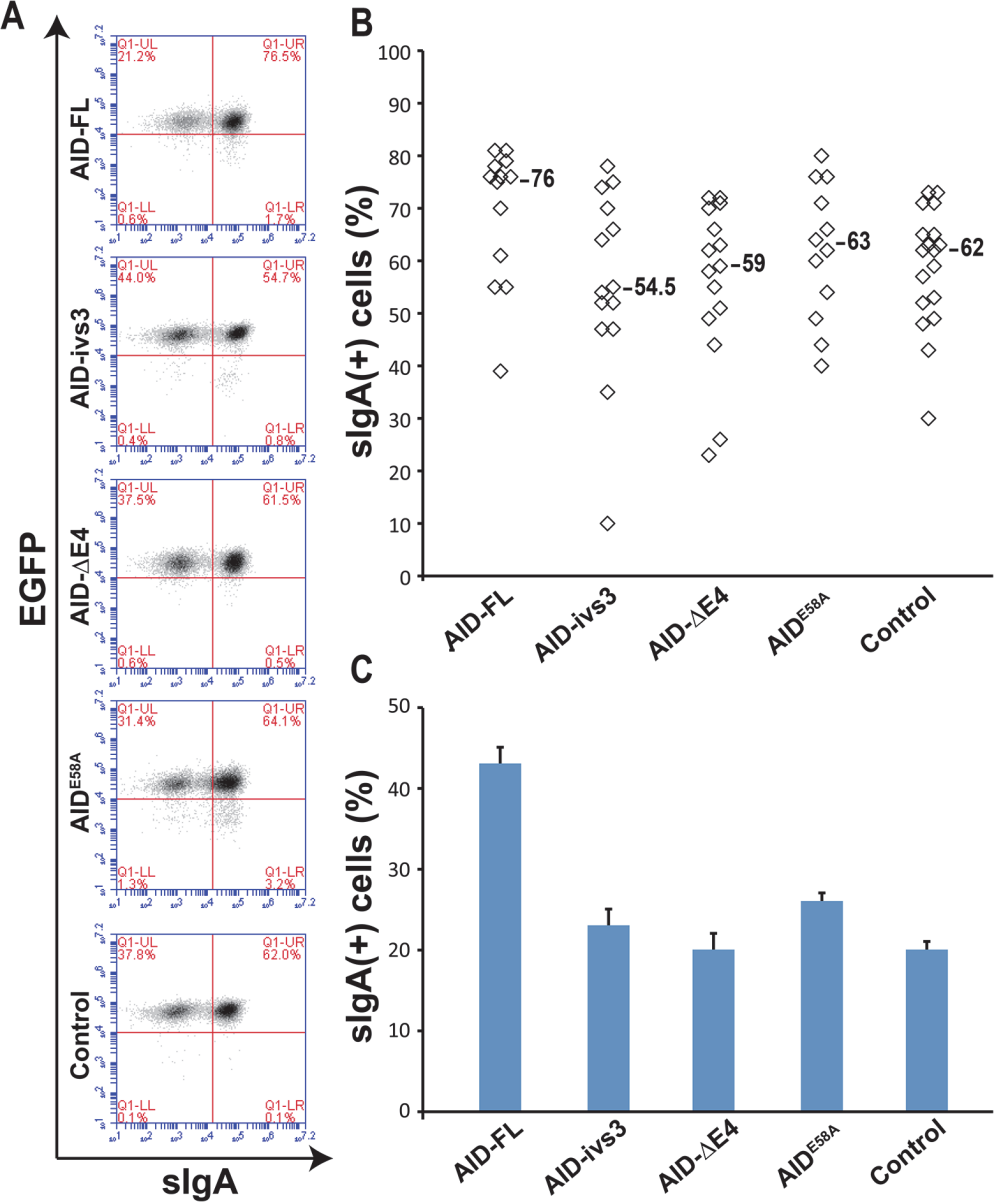
AID variants do not affect Class Switch Recombination in CH12F3 cells. (A) FACS profiles of representative CH12F3 clones stably expressing AID-FL, AID-ivs3, AID-ΔE4 AID^E58A^, or an EGFP negative control. Cells were induced for CSR and assayed by FACS after 72 hr. The numbers in the upper-right quadrants indicate the percentage of cells within the scatter window that are sIgA(+)/EGFP(+). (B) Each datapoint in the plot represents the percentage of sIgA(+)/EGFP(+) cells in an independent clone expressing the various constructs. While there is an increase in the efficiency of the CSR in the clones transfected with full-length AID, none of the samples reaches statistical significance (one-way ANOVA coupled with Tukey’s test). The median level of CSR is indicated. (C) CSR analysis of cells transiently transfected with the constructs encoding for AID FL, AID-ΔE4 and AID-ivs3, AID-E58A, and a plasmid containing only the EGFP negative control. The analysis was performed on the bulk of transfected cells after 48 hours of selection with puromycin and 72 hours after induction with the stimulation cocktail. Transfected EGFP(+) cells represented ~2% of the total population. The only sample affecting the CSR is the one expressing the full-length AID (one-way ANOVA coupled with Tukey’s test, *P = 0*.*0004*). The histograms represent the mean of four different experiments and the standard error is shown.

To test whether any of these constructs could modulate the activity of the induced endogenous AID, we have induced CSR in the selected clones with TGF-ß, IL-4 and CD40 stimulation. Cells were assayed 72 hours after stimulation, a timepoint that usually provides high levels of CSR (~50%). Indeed, only overexpression of the full-length AID elicited a slight increase (not statistically significant) in the efficiency of CSR. All other constructs showed levels of CSR very similar to those of the controls (Fig. 4A, B). Similar results were obtained analyzing the EGFP(+) population from transiently transfected CH12F3 cells (Fig. 4C). Thus, our data suggest that overexpression of the catalytically inactive natural isoforms of AID is not able to modulate the activity of the endogenous AID, at least with regards to the Class Switch Recombination process.

## Conclusions

A number of splice variants of AID have been identified in normal B cells and in lymphoproliferative diseases beyond the full-length one [27–36], and their presence correlated to the prognosis of the disease [27,29–31,35]. However, their functional significance is controversial [34,39]. The absence of catalytic activity ([39], our data) suggests that—if translated—their effect might be indirect, through their interplay with the pathways involved in the antigen-driven antibody diversification processes. Indeed, our results exclude that coexpression with endogenous AID substantially alters the efficiency of CSR (as opposed to the overexpression of full-length AID). Our results do not rule out the possibility that these alternatively spliced isoforms of AID could still play a role, either as transcripts or simply because their transcription competes with that of the full-length AID.

## Supporting Information

S1 File Unprocessed Western data. The compressed file contains the ChemiDoc files of the western blots shown in Figs. 1B and 2.
(ZIP)

## References

[1] Muramatsu M, Kinoshita K, Fagarasan S, Yamada S, Shinkai Y, Honjo T. Class switch recombination and hypermutation require activation-induced cytidine deaminase (AID), a potential RNA editing enzyme. Cell. 2000; 102: 553–563. 11007474 10.1016/s0092-8674(00)00078-7

[2] Petersen-Mahrt SK, Harris RS, Neuberger MS. AID mutates E. coli suggesting a DNA deamination mechanism for antibody diversification. Nature. 2002; 418: 99–103. 12097915 10.1038/nature00862

[3] Neuberger MS. Antibody diversification by somatic mutation: from Burnet onwards. Immunol Cell Biol. 2008; 86: 124–132. 10.1038/sj.icb.7100160 18180793

[4] Pasqualucci L, Neumeister P, Goossens T, Nanjangud G, Chaganti RS, et al Hypermutation of multiple proto-oncogenes in B-cell diffuse large-cell lymphomas. Nature. 2001; 412: 341–346. 11460166 10.1038/35085588

[5] Kotani A, Kakazu N, Tsuruyama T, Okazaki IM, Muramatsu M, et al Activation-induced cytidine deaminase (AID) promotes B cell lymphomagenesis in Emu-cmyc transgenic mice. PNAS. 2007; 104: 1616–1620. 17251349 10.1073/pnas.0610732104 PMC1785248

[6] Okazaki IM, Kotani A, Honjo T. Role of AID in Tumorigenesis. Adv Immunol. 2007; 94: 245–273. 17560277 10.1016/S0065-2776(06)94008-5

[7] Liu M, Duke JL, Richter DJ, Vinuesa CG, Goodnow CC, et al Two levels of protection for the B cell genome during somatic hypermutation. Nature. 2008; 451: 841–845. 10.1038/nature06547 18273020

[8] Ramiro AR, Jankovic M, Eisenreich T, Difilippantonio S, Chen-Kiang S, et al AID is required for c-myc/IgH chromosome translocations in vivo. Cell. 2004; 118: 431–438. 15315756 10.1016/j.cell.2004.08.006

[9] Wang CL, Harper RA, Wabl M. Genome-wide somatic hypermutation. PNAS. 2004; 101: 7352–7356. 15123833 10.1073/pnas.0402009101 PMC409922

[10] Parsa JY, Basit W, Wang CL, Gommerman JL, Carlyle JR, Martin A. AID mutates a non-immunoglobulin transgene independent of chromosomal position. Mol Immunol. 2007; 44: 567–575. 16542725 10.1016/j.molimm.2006.02.003

[11] Dorsett Y, Robbiani DF, Jankovic M, Reina-San-Martin B, Eisenreich TR, Nussenzweig MC. A role for AID in chromosome translocations between c-myc and the IgH variable region. J Exp Med. 2007; 204: 2225–2232. 17724134 10.1084/jem.20070884 PMC2118712

[12] Robbiani DF, Bothmer A, Callen E, Reina-San-Martin B, Dorsett Y, et al AID Is Required for the Chromosomal Breaks in c-myc that Lead to c-myc/IgH Translocations. Cell. 2008; 135: 1028–1038. 10.1016/j.cell.2008.09.062 19070574 PMC2713603

[13] Robbiani DF, Nussenzweig MC. Chromosome translocation, B cell lymphoma, and activation-induced cytidine deaminase. Annu Rev Pathol. 2013; 8: 79–103. 10.1146/annurev-pathol-020712-164004 22974238

[14] Yadav A, Olaru A, Saltis M, Setren A, Cerny J, Livák F. Identification of a ubiquitously active promoter of the murine activation-induced cytidine deaminase (AICDA) gene. Mol Immunol. 2006; 43: 529–541. 16005067 10.1016/j.molimm.2005.05.007

[15] Tran TH, Nakata M, Suzuki K, Begum NA, Shinkura R, et al B cell-specific and stimulation-responsive enhancers derepress Aicda by overcoming the effects of silencers. Nat Immunol. 2010; 11: 148–154. 10.1038/ni.1829 19966806

[16] Ta VT, Nagaoka H, Catalan N, Durandy A, Fischer A, et al AID mutant analyses indicate requirement for class-switch-specific cofactors. Nat Immunol. 2003; 4: 843–848. 12910268 10.1038/ni964

[17] Ito S, Nagaoka H, Shinkura R, Begum N, Muramatsu M, et al Activation-induced cytidine deaminase shuttles between nucleus and cytoplasm like apolipoprotein B mRNA editing catalytic polypeptide 1. PNAS. 2004; 101: 1975–1980. 14769937 10.1073/pnas.0307335101 PMC357037

[18] McBride KM, Barreto V, Ramiro AR, Stavropoulos P, Nussenzweig MC. Somatic hypermutation is limited by CRM1-dependent nuclear export of activation-induced deaminase. J Exp Med. 2004; 199: 1235–1244. 15117971 10.1084/jem.20040373 PMC2211910

[19] Brar SS, Watson M, Diaz M. Activation-induced cytosine deaminase (AID) is actively exported out of the nucleus but retained by the induction of DNA breaks. J Biol Chem. 2004; 279: 26395–26401. 15087440 10.1074/jbc.M403503200

[20] Patenaude AM, Orthwein A, Hu Y, Campo VA, Kavli B, et al Active nuclear import and cytoplasmic retention of activation-induced deaminase. Nat Struct Mol Biol. 2009; 16: 517–527. 10.1038/nsmb.1598 19412186

[21] Orthwein A, Patenaude AM, Affar EB, Lamarre A, Young JC, Di Noia JM. Regulation of activation-induced deaminase stability and antibody gene diversification by Hsp90. J Exp Med. 2010; 207: 2751–2765. 10.1084/jem.20101321 21041454 PMC2989769

[22] Ellyard JI, Benk AS, Taylor B, Rada C, Neuberger MS. The dependence of Ig class-switching on the nuclear export sequence of AID likely reflects interaction with factors additional to Crm1 exportin. Eur J Immunol. 2011; 41: 485–490. 10.1002/eji.201041011 21268017 PMC3437479

[23] Uchimura Y, Barton LF, Rada C, Neuberger MS. REG-γ associates with and modulates the abundance of nuclear activation-induced deaminase. J Exp Med. 2011; 208: 2385–2391. 10.1084/jem.20110856 22042974 PMC3256965

[24] Basu U, Chaudhuri J, Alpert C, Dutt S, Ranganath S, et al The AID antibody diversification enzyme is regulated by protein kinase A phosphorylation. Nature. 2005; 438: 508–511. 16251902 10.1038/nature04255

[25] McBride KM, Gazumyan A, Woo EM, Barreto VM, Robbiani DF, et al Regulation of hypermutation by activation-induced cytidine deaminase phosphorylation. PNAS. 2006; 103: 8798–8803. 16723391 10.1073/pnas.0603272103 PMC1482658

[26] Pasqualucci L, Kitaura Y, Gu H, Dalla-Favera R. PKA-mediated phosphorylation regulates the function of activation-induced deaminase (AID) in B cells. PNAS. 2006; 103: 395–400. 16387847 10.1073/pnas.0509969103 PMC1326186

[27] Albesiano E, Messmer BT, Damle RN, Allen SL, Rai KR, Chiorazzi N. Activation-induced cytidine deaminase in chronic lymphocytic leukemia B cells: expression as multiple forms in a dynamic, variably sized fraction of the clone. Blood. 2003; 102: 3333–3339. 12855567 10.1182/blood-2003-05-1585

[28] Greeve J, Philipsen A, Krause K, Klapper W, Heidorn K, et al Expression of activation-induced cytidine deaminase in human B-cell non-Hodgkin lymphomas. Blood. 2003; 101: 3574–3580. 12511417 10.1182/blood-2002-08-2424

[29] Oppezzo P, Vuillier F, Vasconcelos Y, Dumas G, Magnac C, et al Chronic lymphocytic leukemia B cells expressing AID display dissociation between class switch recombination and somatic hypermutation. Blood. 2003; 101: 4029–4032. 12521993 10.1182/blood-2002-10-3175

[30] McCarthy H, Wierda WG, Barron LL, Cromwell CC, Wang J, et al High expression of activation-induced cytidine deaminase (AID) and splice variants is a distinctive feature of poor-prognosis chronic lymphocytic leukemia. Blood. 2003; 101: 4903–4908. 12586616 10.1182/blood-2002-09-2906

[31] Babbage G, Garand R, Robillard N, Zojer N, Stevenson FK, Sahota SS. Mantle cell lymphoma with t(11;14) and unmutated or mutated VH genes expresses AID and undergoes isotype switch events. Blood. 2004; 103: 2795–2798. 14551145 10.1182/blood-2003-05-1632

[32] Heintel D, Kroemer E, Kienle D, Schwarzinger I, Gleiss A, et al High expression of activation-induced cytidine deaminase (AID) mRNA is associated with unmutated IGVH gene status and unfavourable cytogenetic aberrations in patients with chronic lymphocytic leukaemia. Leukemia. 2004; 18: 756–762. 14961036 10.1038/sj.leu.2403294

[33] Forconi F, Sahota SS, Raspadori D, Ippoliti M, Babbage G, et al Hairy cell leukemia: at the crossroad of somatic mutation and isotype switch. Blood. 2004; 104: 3312–3317. 15284115 10.1182/blood-2004-03-0950

[34] Wu X, Darce JR, Chang SK, Nowakowski GS, Jelinek DF. Alternative splicing regulates activation-induced cytidine deaminase (AID): Implications for suppression of AID mutagenic activity in normal and malignant B-cells. Blood. 2008; 112: 4675–4682. 10.1182/blood-2008-03-145995 18684869 PMC2597133

[35] Marantidou F, Dagklis A, Stalika E, Korkolopoulou P, Saetta A, et al Activation-induced cytidine deaminase splicing patterns in chronic lymphocytic leukemia. Blood Cells Mol Dis. 2010; 44: 262–267. 10.1016/j.bcmd.2009.12.005 20117026

[36] Palacios F, Moreno P, Morande P, Abreu C, Correa A, et al High expression of AID and active class switch recombination might account for a more aggressive disease in unmutated CLL patients: link with an activated microenvironment in CLL disease. Blood. 2010; 115: 4488–4496. 10.1182/blood-2009-12-257758 20233972

[37] Noguchi E, Shibasaki M, Inudou M, Kamioka M, Yokouchi Y, et al Association between a new polymorphism in the activation-induced cytidine deaminase gene and atopic asthma and the regulation of total serum IgE levels. J Allergy Clin Immunol. 2001; 108: 382–386. 11544457 10.1067/mai.2001.117456

[38] Iacobucci I, Lonetti A, Messa F, Ferrari A, Cilloni D, et al Different isoforms of the B-cell mutator activation-induced cytidine deaminase are aberrantly expressed in BCR-ABL1-positive acute lymphoblastic leukemia patients. Leukemia. 2010; 24: 66–73. 10.1038/leu.2009.197 19759560

[39] van Maldegem F, Scheeren FA, Aarti Jibodh R, Bende RJ, Jacobs H, van Noesel CJ. AID splice variants lack deaminase activity. Blood. 2009; 113: 1862–4; author reply 1864. 10.1182/blood-2008-08-175265 19228934

[40] van Maldegem F, Jibodh RA, van Dijk R, Bende RJ, van Noesel CJ. Activation-Induced Cytidine Deaminase Splice Variants Are Defective Because of the Lack of Structural Support for the Catalytic Site. J Immunol. 2010; 184: 2487–2491. 10.4049/jimmunol.0903102 20118283

[41] Nakamura M, Kondo S, Sugai M, Nazarea M, Imamura S, Honjo T. High frequency class switching of an IgM+ B lymphoma clone CH12F3 to IgA+ cells. Int Immunol. 1996; 8: 193–201. 8671604 10.1093/intimm/8.2.193

[42] Arakawa H, Hauschild J, Buerstedde JM. Requirement of the activation-induced deaminase (AID) gene for immunoglobulin gene conversion. Science. 2002; 295: 1301–1306. 11847344 10.1126/science.1067308

[43] DuBridge RB, Tang P, Hsia HC, Leong PM, Miller JH, Calos MP. Analysis of mutation in human cells by using an Epstein-Barr virus shuttle system. Mol Cell Biol. 1987; 7: 379–387. 3031469 10.1128/mcb.7.1.379-387.1987 PMC365079

[44] Conticello SG, Ganesh K, Xue K, Lu M, Rada C, Neuberger MS. Interaction between antibody-diversification enzyme AID and spliceosome-associated factor CTNNBL1. Mol Cell. 2008; 31: 474–484. 10.1016/j.molcel.2008.07.009 18722174

[45] Rebhandl S, Huemer M, Zaborsky N, Gassner FJ, Catakovic K, et al Alternative splice variants of AID are not stoichiometrically present at the protein level in chronic lymphocytic leukemia. Eur J Immunol. 2014; 44: 2175–2187. 10.1002/eji.201343853 24668151 PMC4209801

